# The mitochondrial genome sequence of the ciliate *Paramecium caudatum *reveals a shift in nucleotide composition and codon usage within the genus *Paramecium*

**DOI:** 10.1186/1471-2164-12-272

**Published:** 2011-05-31

**Authors:** Dana Barth, Thomas U Berendonk

**Affiliations:** 1 University of Leipzig, Chair of Molecular Evolution and Animal Systematics, Talstrasse 33, 04103 Leipzig, Germany; 2 Dresden University of Technology, Institute for Hydrobiology, Zellescher Weg 40, 01062 Dresden, Germany

## Abstract

**Background:**

Despite the fact that the organization of the ciliate mitochondrial genome is exceptional, only few ciliate mitochondrial genomes have been sequenced until today. All ciliate mitochondrial genomes are linear. They are 40 kb to 47 kb long and contain some 50 tightly packed genes without introns. Earlier studies documented that the mitochondrial guanine + cytosine contents are very different between *Paramecium tetraurelia *and all studied *Tetrahymena *species. This raises the question of whether the high mitochondrial G+C content observed in *P. tetraurelia *is a characteristic property of *Paramecium *mtDNA, or whether it is an exception of the ciliate mitochondrial genomes known so far. To test this question, we determined the mitochondrial genome sequence of *Paramecium caudatum *and compared the gene content and sequence properties to the closely related *P. tetraurelia*.

**Results:**

The guanine + cytosine content of the *P. caudatum *mitochondrial genome was significantly lower than that of *P. tetraurelia *(22.4% vs. 41.2%). This difference in the mitochondrial nucleotide composition was accompanied by significantly different codon usage patterns in both species, i.e. within *P. caudatum *clearly A/T ending codons dominated, whereas for *P. tetraurelia *the synonymous codons were more balanced with a higher number of G/C ending codons. Further analyses indicated that the nucleotide composition of most members of the genus *Paramecium *resembles that of *P. caudatum *and that the shift observed in *P. tetraurelia *is restricted to the *P. aurelia *species complex.

**Conclusions:**

Surprisingly, the codon usage bias in the *P. caudatum *mitochondrial genome, exemplified by the effective number of codons, is more similar to the distantly related *T. pyriformis *and other single-celled eukaryotes such as *Chlamydomonas*, than to the closely related *P. tetraurelia*. These differences in base composition and codon usage bias were, however, not reflected in the amino acid composition. Most probably, the observed picture is best explained by a hitherto unknown (neutral or adaptive) mechanism that increased the guanine + cytosine content in *P. tetraurelia *mtDNA on the one hand, and strong purifying selection on the ancestral amino acid composition on the other hand. These contradicting forces are counterbalanced by a considerably altered codon usage pattern.

## Background

The genomic information and evolution of the mitochondrial genomes of metazoans is well documented. Knowledge on the evolution of mitochondrial DNA (mtDNA) in single-celled organisms on the other hand is more scarce. In order to promote the understaning of the mitochondrial genome evolution of single-celled eukaryotes, it is necessary to fill the large existing gaps of knowledge and data. For example only few ciliate mitochondrial genomes have been sequenced until today, most of them belonging to the genus *Tetrahymena*: *T. pyriformis *[1], *T. thermophila *[2], *T. pigmentosa*, *T. malacensis, T. paravorax *[3]. While the newest sequence belongs to *Euplotes minuta *[4], *P. tetraurelia *has been among the first unicellular eukaryotes, for which the complete mitochondrial genome sequence was determined [5]. Therefore, to fill in one of the above mentioned gaps, we decided to sequence the mitochondrial genome of *P. caudatum*, a close relative of the *P. aurelia *species complex, in order to elucidate the mitochondrial genome evolution within this important ciliate genus.

Two interesting aspects of ciliate mtDNA are predominant and have made the assembly of mitochondrial genomes difficult. First, all hitherto known ciliate mitochondrial genomes are linear, from 40 kb (*P. tetraurelia*) to 47 kb (*Tetrahymena*) long, and contain some 50 tightly packed genes without introns. Second, for about half of the open reading frames (ORFs) in the ciliate mtDNA, a definite protein function is unknown, because there is not enough similarity to known proteins in other organisms [1,2]. Among these ORFs, *ymf77 *in the *Tetrahymena *mtDNA is particularly unusual. This gene is ~1,350 amino acid residues long, shows extreme sequence divergence among *Tetrahymena *species and has no homolog in the *P. tetraurelia *or *Euplotes *mitochondrial genomes.

*Paramecium *and *Tetrahymena *belong to the class Oligohymenophorea, one out of eleven lineages within the phylum Ciliophora. Despite similarities in gene content and genome organization in *P. tetraurelia *and *Tetrahymena *mtDNA, there are great differences in the nucleotide composition. All studied *Tetrahymena *mitochondrial genomes have a guanine and cytosine (G+C) content of ~20%, whereas in *P. tetraurelia*, the G+C content is more than twice as high (c. 41%, [1]). *Paramecium tetraurelia *and *T. thermophila *are model organisms, whose macronuclear genome sequences have been recently completed [6,7]. Interestingly, the nuclear G+C contents are not significantly different in both species (69.9% vs. 72.3%, [8]). This raises the question of whether the high mitochondrial G+C content observed in *P. tetraurelia *is a characteristic property of *Paramecium *mtDNA, or an exception of the ciliate mitochondrial genomes known so far.

To test this question, we determined the mitochondrial genome sequence of *P. caudatum *and compared its gene content and sequence properties to the closely related *P. tetraurelia*.

## Results

### General characterization

**Figure 1:**
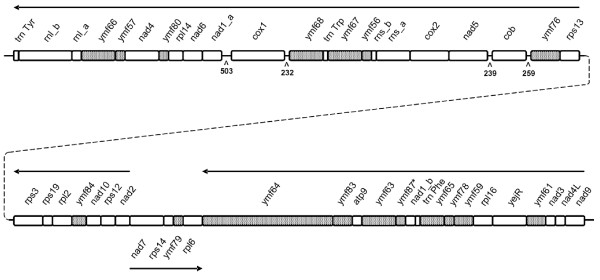
**Gene map of the linear mitochondrial genome of *Paramecium caudatum***. The transcriptional orientation of the genes is indicated by arrows. Putative protein-coding genes with unknown function (ORFs) are displayed in grey and the *P. caudatum *specific ORF *ymf87 *is marked with an asterisk. Intergenic spacers of >100 nucleotides are also shown, the numbers represent spacer lengths in bp.

The mitochondrial genome of *Paramecium caudatum *has been described as a linear molecule of 40-44 kb [9]. We determined 43,660 bp of the genome sequence [GenBank:FN424190], excluding only the terminal repeat regions that usually flank the linear ciliate mtDNA. The overall nucleotide composition was 9.9% G, 42.7% A, 34.9% T, and 12.5% C, the G+C content thereby being significantly lower than that of *P. tetraurelia *mtDNA (22.4% vs. 41.2%). The coding part of the genome was 41,091 bp (94.1%), the protein-coding part 36,585 bp (83.8%) in length. We determined 25 protein-coding genes with known function, 17 additional ORFs with unknown function, the small and large subunits of the ribosomal RNA genes and three transfer RNA genes (Figure 1). Genes in the *P. caudatum *mitochondrial genome were densely packed, with short intergenic spacers (0-85 bp). However, the intergenic regions adjacent to the *cox1 *and *cob *genes were considerably longer (up to 503 bp; Figure 1). An overview of all protein-coding genes and a comparison with the *P. tetraurelia *mtDNA is given in Table 1.

**Table 1:** Size of protein-coding genes in *P. caudatum *mtDNA and comparative data calculated for *P. caudatum *and *P. tetraurelia*

gene	length (in aa)	sequence identity	Ka/Ks	ORF	length (in aa)	sequence identity	Ka/Ks
*cob*	442	0.91	0.012	*ymf56*	81	0.73	0.067
*cox1*	745	0.93	0.019	*ymf57*	105	0.77	0.069
*cox2*	577	0.84	0.022	*ymf59*	127	0.40	0.340
*atp9*	75	1.00	0.001	*ymf61*	189	0.40	0.298
*yejR*^a^	447	0.47	0.242	*ymf63*^a^	525	0.44	0.258
*nad1_a*	287	0.93	0.012	*ymf64*^a^	1712	0.56	0.168
*nad1_b*	63	0.71	0.027	*ymf65*^b^	365	-	-
*nad2*	167	0.67	0.093	*ymf66*^b^	412	-	-
*nad3*	120	0.83	0.033	*ymf67*^b^	354	-	-
*nad4*	501	0.70	0.062	*ymf68*	395	0.76	0.044
*nad4L*	115	0.64	0.070	*ymf76*^b^	399	-	-
*nad5*	597	0.75	0.049	*ymf78*	69	0.48	0.298
*nad6*	256	0.73	0.064	*ymf79*	73	0.38	0.323
*nad7*	423	0.91	0.010	*ymf80*	97	0.68	0.076
*nad9*	184	0.67	0.072	*ymf83*	168	0.55	0.206
*nad10*	154	0.95	0.006	*ymf84*	158	0.33	0.291
*rps3*^a^	363	0.57	0.195	*ymf87*^c^	107	-	-
*rps12*	139	0.84	0.024				
*rps13*	244	0.66	0.049				
*rps14*	102	0.59	0.104				
*rps19*^d^	88	0.46	0.188				
*rpl2*	265	0.68	0.523				
*rpl6*	178	0.71	0.055				
*rpl14*	119	0.75	0.030				
*rpl16*	166	0.74	0.047				

The sequence identity values were calculated from amino acid sequences. The Ka/Ks ratio was estimated using the method of Goldman and Yang [47] as implemented in the program Ka/Ks_Calculator [28].

^a ^*yejR*, *rps3, ymf63 *and *ymf64 *were considerably longer in the mtDNA of *P. caudatum*. For comparative analyses the sequences were adjusted to the gene length in *P. tetraurelia*.

^b ^*ymf65*, *ymf66*, *ymf67 *and *ymf76 *each appear to be equivalent to two adjacent ORFs in *P. tetraurelia*. Comparative data are omitted.

^c ^*ymf87 *was only found in *P. caudatum*

^d ^*rps19 *in *P. tetraurelia *was annotated during the present work

The amino acid sequences of genes with known function were more similar between *Paramecium *species than sequences of the putative ORFs, with *atp9 *and *nad10 *being the most conserved genes (Table 1). The ratio of nonsynonymous vs. synonymous substitutions (Ka/Ks) also differed, with Ka/Ks values being significantly higher in putative ORFs than in known genes (Mann-Whitney-U-test z = -3.30; p < 0.001; mean values of 0.203 and 0.080, respectively). An exception was the *rpl2 *gene, which possessed the highest Ka/Ks value among all protein-coding genes in *Paramecium *mtDNA, although it was still well within the range indicative for purifying selection (Table 1).

### Nucleotide composition and codon usage

Nucleotide composition and codon usage of the newly determined *P. caudatum *mitochondrial genome were compared to the previously published mitochondrial genomes of *P. tetraurelia *[GenBank:NC001324] and *T. pyriformis *[GenBank:NC000862]. In *P. caudatum*, the G+C content of the protein-coding genes was similar to the G+C content of *T. pyriformis *(21.7% and 20.2%, respectively), but much lower than that of *P. tetraurelia *(43.2%). This difference was even more pronounced at 3^rd ^codon positions, with 56.5% G or C ending codons in *P. tetraurelia *and only 12.8% and 11.9% G or C ending codons in *P. caudatum *and *T. pyriformis*, respectively. The G+C content of the noncoding regions (intergenic spacers) was slightly lower compared to protein-coding genes in all species, but the relative differences between species were similar (Table 2). In the mitochondrial rRNA genes, on the other hand, the G+C content differed only slightly among the ciliates (Table 2).

**Table 2:** Nucleotide composition in coding and noncoding regions of ciliate mitochondrial genomes

	Protein-coding genes			noncoding DNA			rRNA genes		
	***T. p***.	***P. c***.	***P. t***.	***T. p***.	***P. c***.	***P. t***.	***T. p***.	***P. c***.	***P. t***.
G	10.3	12.1	19.5	6.2	8.0	18.7	13.8	19.0	21.2
A	41.1	35.5	24.8	40.8	35.7	24.3	35.3	35.6	33.0
T	38.7	42.8	32.0	46.3	49.0	36.1	35.7	32.3	30.3
C	9.9	9.6	23.7	6.7	7.3	20.9	15.2	13.1	15.5
**G+C**	**20.2**	**21.7**	**43.2**	**12.9**	**15.3**	**39.6**	**29.0**	**32.1**	**36.7**

*T. p. Tetrahymena pyriformis *[GenBank:NC000862], *P. c. Paramecium caudatum *[GenBank:FN424190], *P. t. Paramecium tetraurelia *[GenBank:NC001324], values are given in percent.

**Figure 2:**
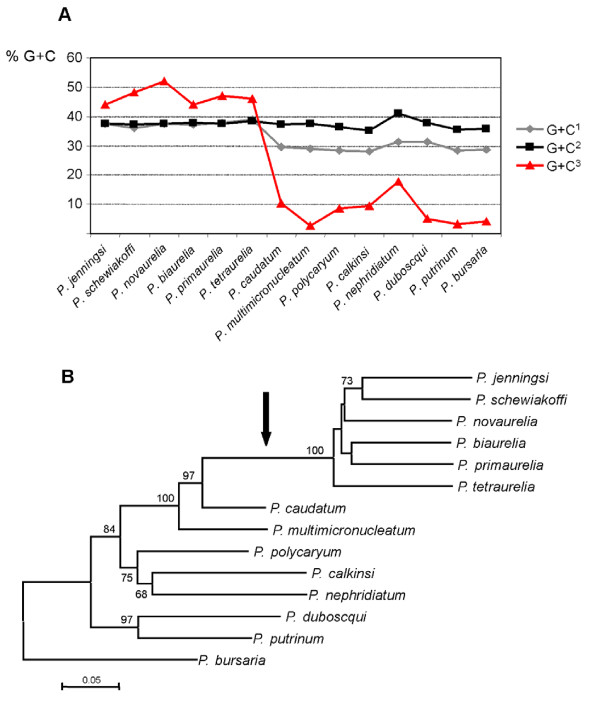
**Mitochondrial nucleotide composition within the genus *Paramecium***. A) Guanine + cytosine content at 1^st^, 2^nd ^and 3^rd ^codon positions of the mitochondrial *cox1 *gene. B) Phylogenetic tree based on 776 bp of the *cox1 *gene. The numbers represent bootstrap values for 2,000 pseudoreplicates in the Neighbor Joining analysis and the scale bar equals 0.05 substitutions/site. The arrow indicates the phylogenetic position of the G+C shift.

In order to gain further insight into the nucleotide composition of the *Paramecium *mtDNA, we sequenced a 776 bp fragment of the Cytochrome oxidase subunit 1 gene (*cox1*) from 13 more *Paramecium *species. A phylogenetic tree of *Paramecium *based on sequences of the *cox1 *gene is shown in Figure 2B. The analysis of the nucleotide composition at 3^rd ^codon positions of the *cox1 *gene revealed high G+C values (45-50%) in species of the *P. aurelia *complex and a low G+C content (3-17%) in the remaining paramecia (Figure 2A).

The codon usage bias (CUB) differed significantly between *P. caudatum *and *P. tetraurelia *(Table 3). Only in one out of 19 amino acids (Phe) the same codon (TTT) was the most frequently used synonymous codon in *P. caudatum *and *P. tetraurelia*, whereas *P. caudatum *and *T. pyriformis *shared the most frequently used codon in 16 amino acids. The type of preferred codons was related to the nucleotide composition of the respective species (Table 2, 3). Generally, for *P. caudatum *and *T. pyriformis *A/T ending synonymous codons were abundant. This was also true for the stop codons, as only TAA was detected to terminate protein-coding genes in *P. caudatum *and *Tetrahymena*, whereas TAG never occurred (Table 3). In *P. tetraurelia *mtDNA, on the other hand, the codon usage was shifted against G/C ending codons. Only in two amino acids (Phe and Trp) the most frequent synonymous codon ended in A or T (and these are the termination codons). Furthermore, both the number of rare codons and the effective number of codons (*Nc*) indicate a more balanced usage of synonymous codons in *P. tetraurelia *compared to the other ciliates (Table 3). A higher *Nc *in *P. tetraurelia *was found for all protein-coding genes and putative ORFs, except *nad9 *(additional file 1 Table S1).

**Table 3:** Comparative codon usage in the mtDNA of *T. pyriformis*, *P. caudatum *and *P. tetraurelia*

		***T***. ***p***.	***P***. ***c***.	***P***. ***t***.			***T***. ***p***.	***P***. ***c***.	***P***. ***t***.
Ala	GCG	*0.03*	*0.03*	*0.08*	Pro	CCG	*0.07*	*0.03*	*0.03*
	GCA	0.32	0.41	0.20		CCA	0.36	0.42	0.16
	GCT	**0.57**	**0.54**	0.30		CCT	**0.54**	**0.51**	0.27
	GCC	*0.07*	*0.02*	**0.41**		CCC	*0.03*	*0.04*	**0.54**
									
Cys	TGT	**0.86**	**0.85**	0.24	Gln	CAG	*0.06*	0.14	**0.58**
	TGC	0.14	0.15	**0.76**		CAA	**0.94**	**0.86**	0.42
									
Asp	GAT	**0.81**	**0.81**	0.36	Arg	AGG	*0.02*	0.17	**0.45**
	GAC	0.19	0.19	**0.64**		AGA	**0.95**	**0.74**	0.16
						CGG	*0.00*	*0.00*	*0.03*
Glu	GAG	*0.08*	0.17	**0.70**		CGA	*0.00*	*0.02*	0.11
	GAA	**0.92**	**0.83**	0.30		CGT	*0.02*	*0.06*	*0.06*
						CGC	*0.00*	*0.01*	0.18
Phe	TTT	**0.89**	**0.95**	**0.62**					
	TTC	0.11	*0.05*	0.38	Ser	AGT	0.21	0.24	*0.07*
						AGC	*0.06*	0.09	**0.27**
Gly	GGG	*0.03*	*0.05*	0.27		TCG	*0.03*	*0.03*	0.12
	GGA	0.16	0.19	0.20		TCA	0.32	0.20	0.10
	GGT	**0.78**	**0.73**	0.16		TCT	**0.34**	**0.42**	0.24
	GGC	*0.03*	*0.04*	**0.37**		TCC	*0.04*	*0.03*	0.20
									
His	CAT	**0.69**	**0.69**	0.31	Thr	ACG	*0.01*	*0.03*	0.22
	CAC	0.31	0.31	**0.69**		ACA	**0.53**	0.45	0.18
						ACT	0.41	**0.49**	0.23
Ile	ATA	**0.62**	0.42	0.21		ACC	*0.06*	*0.03*	**0.37**
	ATT	0.33	**0.53**	0.33					
	ATC	*0.06*	*0.05*	**0.46**	Val	GTG	*0.04*	*0.06*	0.19
						GTA	**0.49**	0.35	0.20
Lys	AAG	*0.04*	0.11	**0.64**		GTT	0.42	**0.55**	0.30
	AAA	**0.96**	**0.89**	0.36		GTC	*0.06*	*0.04*	**0.31**
									
Leu	TTG	*0.03*	0.09	0.13	Trp	TGG	*0.03*	*0.01*	0.48
	TTA	**0.75**	**0.74**	0.14		TGA	**0.97**	**0.99**	**0.52**
	CTG	*0.01*	*0.01*	0.09					
	CTA	0.13	*0.05*	0.15	Tyr	TAT	**0.82**	**0.79**	0.38
	CTT	*0.07*	0.11	0.19		TAC	0.18	0.21	**0.62**
	CTC	*0.01*	*0.01*	**0.31**					
					Stop	TAG	*0.00*	*0.00*	0.39
Met	ATG	1.00	1.00	1.00		TAA	**1.00**	**1.00**	**0.61**
									
Asn	AAT	**0.79**	**0.77**	0.34		*rare*	*28*	*23*	*5*
	AAC	0.21	0.23	**0.66**		*Nc*	31.5	33.5	52.1

**Bold **values indicate the most frequent synonymous codons for each amino acid and species, *italic *values indicate rare codons. The overall number of rare codons (*rare*) and the effective number of codons (*Nc*) are also given for each species.

### Gene content and gene order

Gene composition, gene order and transcriptional orientation were essentially the same as in *P. tetraurelia *[1,5]. Most of the few observed differences between *P. caudatum *and *P. tetraurelia *could be attributed to sequencing or annotation errors in the *P. tetraurelia *mtDNA. For example, the small gene *rps19 *was not described in *P. tetraurelia *neither in the original publication nor in the re-annotation paper [1,5]. We determined *rps19 *in the newly sequenced *P. caudatum *mitochondrial genome as well as in *P. tetraurelia *at the same relative position (between *rps3 *and *rpl2*) where it is located in all *Tetrahymena *species [2,3]. The mitochondrial genome of *P. caudatum *contained one ORF (*ymf87*) that was not present in *P. tetraurelia *(Figure 1). Furthermore, we determined five ORFs in *P. caudatum*, each of which seems to be equivalent to two adjacent ORFs in *P. tetraurelia*. Four of these ORFs (*ymf65, ymf66, ymf67 *and *ymf76*) are also present in *Tetrahymena *mtDNA [1]. The fifth ORF refers to the heme maturase gene (*yejR*), which was much larger than *P. tetraurelia yejR *(447 vs. 255 aa), but similar to *Tetrahymena *(512-522 aa) and *Euplotes minuta yejR *(461 aa) [1-4]. In *P. tetraurelia, ymf82 *is located directly downstream to the *yejR *gene [1]. In *P. caudatum*, however, *ymf82 *was not present at this position or anywhere else in the mitochondrial genome (Figure 1). These results could indicate sequencing errors in the *P. tetraurelia *mitochondrial genome, which led to several frameshifts and premature stopcodons in the annotated sequence.

The most striking difference compared to *P. tetraurelia *was *ymf64*, a large open reading frame of 5,136 bp length (Figure 1). The C-terminal part of this ORF showed significant similarity to *ymf64 *of *P. tetraurelia *and *Tetrahymena *(where this ORF is 234 and 330 amino acids long, respectively) and to the c-terminal part of *rps3 *in *Euplotes*. The largest part of the gene, however, consisted of an N-terminal extension. The analysis revealed one transmembrane region from amino acid 1,469 to 1,491, with the alignable C-terminal part (see above) lying inside and the large extension lying outside of the membrane. BLAST searches of this extension detected no sequence similarity with any known sequence in GenBank.

**Figure 3:**
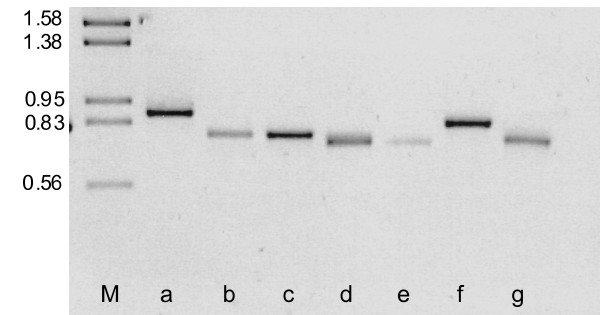
**Tandem repeat region in the open reading frame *ymf64 *amplified from different *Paramecium caudatum *strains**. DNA fragments from strains belonging to the same *cox1 *haplotype PcCOI_a03 [35] were separated on a 2.0% agarose gel. M, DNA size ladder in kb; lanes *a-g*, PCR amplified fragments from the different strains. Number of repeat units: (*a*) 18; (*b*) 11; (*c*) 11; (*d*) 9; (*e*) 9; (*f*) 14; (*g*) 10.

Besides its size, *ymf64 *possessed another unusual feature: an intragenic minisatellite, composed of nine repeat units of 18 bp close to the middle of the ORF. We amplified and sequenced this gene region from 20 further *P. caudatum *strains and found the repeat number to be highly variable, even between strains sharing the same *cox1 *haplotype (Figure 3). We identified 12 length variants with repeat numbers ranging from six to 23 units. No length polymorphism (heteroplasmy) was detected within individual *P. caudatum *strains. The repeat was imperfect as we found differences (substitutions) between the repeat units within one sequence as well as variations between different strains. However, these sequence variations never led to nonsense mutations. Since the repeat unit size was a multiple of three nucleotides, length variation in the repeat region did not cause a frameshift in the gene and left the reading frame intact.

## Discussion

### Codon usage

Closely related species usually show similar nucleotide composition and codon usage patterns. For example, five *Tetrahymena *species possess very similar mitochondrial G+C contents (18.5% - 21.3%; [3]), whereas *T. pyriformis*, *P. tetraurelia *and *E. minuta *differ significantly in this respect (21.7%, 41.9% and 36.0%, respectively; [1,4]). Therefore, it was unexpected that nucleotide composition and codon usage of *P. caudatum *mtDNA were much more similar to *Tetrahymena *than to the congeneric *P. tetraurelia*.

Phylogenetic relationships within the genus *Paramecium *are well studied [10,11]. In a species phylogeny based on 18S rRNA data, *P. caudatum *and the *P. aurelia *species complex are closely related and belong to the "*P. aurelia *subgroup" [11]. This was also supported by the tree based on *cox1 *sequences (Figure 2B). Taken into consideration the phylogenetic position of *P. tetraurelia*, it seems reasonable to hypothesize that a low G+C content and the preference for A/T ending codons represent the ancestral state in the genus *Paramecium*. An increased G+C content in combination with the preference for G/C ending codons would be a derived condition in *P. aurelia *mtDNA. No further complete *Paramecium *mitochondrial genomes were available to test this hypothesis. We analyzed the nucleotide composition in a fragment of the *cox1 *gene, which can be used as an indicator for the overall mitochondrial G+C content [12,13]. These sequences supported the above hypothesis, since the shift in the nucleotide composition obviously took place in the lineage leading to the *P. aurelia *species complex (Figure 2A, B). Of course, this *cox1 *data set contained only few of the 15 known *P. aurelia *species, and for a conclusive statement all relevant species should be included. But in this context, please note that [14] and [15] obtained DNA sequences from different mitochondrial genes of all *P. aurelia *species and these data suggest that the increased mitochondrial G+C content is a consistent pattern throughout the species complex. The observed shift in the nucleotide composition of *P. tetraurelia *mtDNA was associated with a significantly reduced CUB. The CUB in *P. caudatum *mtDNA, exemplified by the effective number of codons, was similar to *T. pyriformis *and other single-celled eukaryotes such as *Chlamydomonas *[16]. *P. tetraurelia*, on the other hand, had a much lower CUB (i.e. higher *Nc*) in nearly all genes (Table 3, additional file 1, Table S1). These differences in base composition and CUB were not reflected in the derived amino acid composition, which was similar for all the compared species (additional file 2, Figure S1). This suggests strong purifying selection on the mitochondrial amino acid composition in *P. tetraurelia*.

The causes for this large shift in G+C content and codon usage in *P. tetraurelia *mtDNA are unknown. In a recent publication, the complete macronuclear genome sequences of *P. tetraurelia *and *T. thermophila *were compared [8]. The authors observed very similar nucleotide composition and codon usage patterns in both species. Thus, whatever caused the shift in the *P. tetraurelia *mitochondrial genome seems to have only affected mtDNA and not nuclear DNA. Furthermore, the altered nucleotide composition was observed for both protein-coding and noncoding regions (Table 2), indicating that the increased G+C content is not the consequence of selection for either a higher expression level of *P. tetraurelia *mitochondrial genes, or towards certain codons. Possible explanations for the increased G+C content in the *P. tetraurelia *mitochondrial genome are discussed below.

A high G+C content could be an adaptation to temperature, UV exposure or other environmental conditions that require a high DNA stability [17] (but see [18]). Although the presently available data suggest that some *P. aurelia *species may be restricted to certain biogeographic regions [19,20], there is no evidence for *P. tetraurelia *generally preferring higher temperatures than *P. caudatum*. In fact, both species often co-occur and have to cope with similar environmental conditions [21].

The observed pattern in *P. tetraurelia *mtDNA could as well be the consequence of neutral processes like biased gene conversion (BGC) or a general change in mutation pressure from AT towards GC. A neutral process being the underlying mechanism is in concordance with the observation that the G+C content in *P. tetraurelia *mtDNA was highest at most neutrally evolving sites (3^rd ^codon positions and noncoding regions). In more constrained mtDNA regions (2^nd ^codon positions and ribosomal RNA genes), the base composition of *P. tetraurelia *was not significantly different from *P. caudatum *(Table 2, Figure 2). However, this observation is not per se evidence for neutrality of the underlying mechanism. It is in principle also consistent with an adaptive process in combination with strong selective pressure on the amino acid composition.

BGC is a GC biased repair process in genome regions that undergo recombination and has been identified as a major drive in genome evolution [22]. Gene conversion has been suggested as recombinational mechanism in the mitochondrial genome of the killifish *Kryptolebias marmoratus *[23], and as an explanation for the exceptionally high mitochondrial G+C content in the green alga *Polytomella capuana *[24]. Previous studies using mtDNA found no evidence for recombination events among *P. aurelia *species [14,15]. It is, nevertheless, possible that such a mechanism has played a role in the evolutionary history of the *P. aurelia *complex.

In this context it is interesting to note, that a whole genome duplication (WGD) has occurred in the common ancestor of the *P. aurelia *complex [6]. A significantly reduced CUB was also found in nuclear encoded mitochondrial genes of *Saccharomyces *species, which experienced a WGD [25]. To our knowledge, the influence of a WGD on the mitochondrial genome has not been investigated. It is tempting to speculate that the WGD influenced the crosstalk between nuclear and mitochondrial genomes (e.g. through nuclear encoded genes for the mitochondrial replication and repair machinery).

In conclusion, the observed picture is best explained by a hitherto unknown (neutral or adaptive) mechanism that increased the G+C content in *P. aurelia *mtDNA on the one hand, and strong purifying selection on the ancestral amino acid composition on the other hand. These contradicting forces are counterbalanced by a considerably altered codon usage pattern.

### Gene content and sequence evolution compared to *P. tetraurelia*

The size of the *P. caudatum *mitochondrial genome was within the range predicted from earlier RFLP analyses [9] and ~10% longer than the mitochondrial genome of *P. tetraurelia *[5]. This difference was primarily due to the presence of the large open reading frame *ymf64 *in *P. caudatum *(discussed in more detail below).

We found one unique ORF (*ymf87*) in *P. caudatum*, which was not present in *P. tetraurelia*. This is in contrast to *Tetrahymena*, where complete synteny among five phylogenetically diverse *Tetrahymena *species was observed, except for some rare gene duplications [3].

The existence of many ORFs of unknown function is a general problem in protist mitochondrial genomes [26]. Likewise, for about half of the protein-coding mitochondrial genes in ciliates no definite function can be assigned [1-3]. One main objective of studies obtaining new ciliate mitochondrial genomes is therefore the annotation of these unknown *ymf *genes. Unfortunately we failed to assign any function to hitherto unknown genes, which is most likely due to the high evolutionary rate of ciliate mtDNA. This problem even challenged the alignment of some ORFs from the closely related *P. caudatum *and *P. tetraurelia*. For example, in *ymf84 *only 33% of the amino acids were identical among both species. Therefore, BLAST searches as well as manual alignments could not determine a possible equivalent of this gene in *Tetrahymena*. The authors of the *T. pyriformis *mtDNA study suggested a possible homology of *Paramecium ymf84 *and *T. pyriformis ymf74 *based on gene size and position (between *nad10 *and *rpl2*) [1]. The recently published mitochondrial genome sequence of the rather distantly related *Euplotes minuta *confirmed this suggestion, as this conserved gene order was also found in *Euplotes *[4].

In general, evolutionary rates are governed by negative (purifying) selection, neutrality or positive (diversifying) selection. The ratio of nonsynonymous and synonymous substitution rates (Ka/Ks) allows an estimation of the selective constraint on a given gene. Values <1 are indicative for purifying selection, whereas values >1 indicate diversifying selection [27,28]. Ka/Ks values of the protein-coding genes in the *Paramecium *mtDNA were generally <1, indicating that purifying selection is acting on all genes.

The genes with high sequence similarities between *P. caudatum *and *P. tetraurelia *also had low Ka/Ks values. This may indicate that the selective optimum for those genes is similar in the compared species. An exception to this trend was the ribosomal protein gene *rpl2*, whose Ka/Ks ratio indicates a relaxed selective constraint in combination with moderate sequence divergence. In *Tetrahymena*, no elevated Ka/Ks ratios were observed for this gene [2,3]. In the present study, putative ORFs had significantly (2.5fold) higher Ka/Ks values compared to known protein-coding genes, indicating a lower selective pressure on those non-annotated genes. Similar results were obtained in a study comparing *T. pyriformis *and *T. thermophila *[2]. Furthermore, these authors supposed that the high divergence of putative ORFs causes difficulties to detect homologous genes through similarity searches. Some of the putative ORFs in *Paramecium*, however, had low Ka/Ks values and a relatively high sequence similarity in *P. caudatum *and *P. tetraurelia *(Table 1). It is surprising that for these genes, which are conserved within ciliates and seem to be under notable selective pressure, no homologs could be detected in other organisms.

### Ymf64

The most conspicuous feature in the *P. caudatum *mtDNA was a large N-terminal extension of *ymf64 *compared to the previously published ciliate mitochondrial genomes. This was also the main reason for the length difference between the mitochondrial genomes of *P. tetraurelia *and *P. caudatum *(40,469 vs. 43,660 bp). An initial PCR survey revealed that the large gene extension was not present in the mitochondrial genomes of three further members of the *P. aurelia *complex (*P. primaurelia*, *P. pentaurelia *and *P. jenningsi*), *P. multimicronucleatum*, *P. nephridiatum *and *P. putrinum *(data not shown). These results together with the phylogenetic analysis (Figure 2B) suggest that this gene extension is a unique feature of *P. caudatum *rather than an ancestral feature that was lost in *P. tetraurelia*. The C-terminal part of *ymf64 *also showed similarity to the C-terminal part of a gene that was annotated as *rps3 *in *Euplotes *(but is not homologous to *rps3 *in *Paramecium *and *Tetrahymena *mtDNA). Interestingly, also in *Euplotes *this gene possesses a large N-terminal extension, although it is smaller than in *P. caudatum *(gene length of 758 aa in *E. minuta*) [4]. The size of *ymf64 *resembles *ymf77 *in the *Tetrahymena *mtDNA, which is approximately 1,300 amino acids long [2]. However, besides the gene length no other similarities could be detected. The existence of two remarkably large but unrelated mitochondrial genes in two relatively closely related ciliate genera is surprising and needs further study. We are currently investigating the sequence and length variation of *ymf64 *in *P. caudatum *and will furthermore apply RT-PCR analyses to test whether the gene is expressed in full length.

The length variable repeat in the middle of *ymf64 *is the first report of an intragenic mitochondrial minisatellite in ciliates. Variable Number Tandem Repeats (VNTRs) or minisatellites are common in the mtDNA of many different organisms, especially metazoans [29]. The high variability (i.e. frequent changes in the number of repeat units) is supposed to occur through slipped strand mispairing during mtDNA replication. In most cases these VNTRs are located in or adjacent to the noncoding mitochondrial control region. To our knowledge, an intragenic mitochondrial minisatellite has been reported only from a gene of unknown function in the oomycete *Phytophthora sojae *[30]. On the other hand, minisatellites encoding nuclear sequences are not unusual. Genes containing polymorphic repeat elements are capable to rapidly adapt to changing environmental conditions or to generate new functions. This is of special importance in developmental genes of multicellular organisms [31] and in cell wall/surface proteins of unicellular prokaryotes [32] and eukaryotes [33] Based on the present data, however, it is not possible to suggest a potential function for the repeat region within *ymf64*.

Minisatellites have been useful for the investigation of population structure and phylogeographic patterns and also for the identification of individual organisms or clones [34]. Only recently, mtDNA sequences have been successfully applied as genetic markers for the investigation of intraspecific variation in ciliates [35,36]. In one of these studies several polymorphic *cox1 *haplotypes were observed among natural isolates of *P. caudatum *and *P. multimicronucleatum*, which reflected no clear geographic structure [35]. The *ymf64 *repeat region proved to be polymorphic in different *P. caudatum *isolates that share the same *cox1 *haplotype (Figure 3). This indicates a higher evolutionary rate of this sequence region. Further analyses including more isolates will show whether the *ymf64 *minisatellite has potential as a genetic marker for *Paramecium*.

## Conclusions

The aim of this study was to compare the mitochondrial genome sequence of *Paramecium caudatum *to the previously published mtDNA of P. *tetraurelia*. While gene order and gene content were very similar in both species, the analysis revealed that nucleotide composition and codon usage bias of the *P. caudatum *mtDNA differed greatly from *P. tetraurelia*. The G+C content of the *P. tetraurelia *mtDNA was nearly twice as high as in *P. caudatum *(41.2% vs. 22.4%) and the codon usage bias was much lower (Nc=: 52.1 vs. 33.5). Unexpectedly, the effective number of codons in the *P. caudatum *mitochondrial genome, is more similar to the distantly related *T. pyriformis *and other single-celled eukaryotes such as *Chlamydomonas*, than to the closely related *P. tetraurelia*. The analysis of *cox1 *data from additional *Paramecium *species showed that the shift towards a higher mitochondrial G+C content took place in the lineage leading to the *P. aurelia *species complex. The reasons for this shift are presently unknown and future studies will investigate whether this is linked to the whole genome duplication that occurred in the common ancestor of the *P. aurelia *complex.

## Methods

### *Paramecium *strains

The *P. caudatum *strain GB-E used for mitochondrial genome sequencing was originally isolated from a small pond in the vicinity of the University of Edinburgh and kept as clonal culture in the collection of the Molecular Evolution laboratory at Leipzig University. The following *Paramecium *species were used for the nucleotide composition analysis and phylogenetic reconstruction (Accession numbers for the *cox1 *sequences are given in parentheses): *P. primaurelia *[GenBank:FN421324], *P. biaurelia *[GenBank:FN421325], *P. tetraurelia *[GenBank:FN421326], *P. novaurelia *[GenBank:FN421327], *P. jenningsi *[GenBank:FN421328], *P. schewiakoffi *[GenBank:AM072773]), *P. multimicronucleatum *[GenBank:AM072766], *P. calkinsi *[GenBank:FN421329], *P. nephridiatum *[GenBank:FN421331], *P. duboscqui *[GenBank:FN421332], *P. polycaryum *[GenBank:FN421330], *P. putrinum *[GenBank:FN421333], *P. bursaria *[GenBank:FN421334].

### Molecular methods

DNA extraction followed a Chelex^® ^protocol as described in [35]. The nearly complete mitochondrial genome of *P. caudatum *was amplified in overlapping fragments of 3-15 kb length using Phusion™ high fidelity DNA polymerase (Finnzymes OY). PCR primers were designed from alignments of conserved gene regions from *P. tetraurelia *[GenBank:NC001324] and *T. pyriformis *[GenBank:NC000862].

PCR products <5 kb were directly sequenced employing a primer walking method. Longer PCR products were gel-purified (GFX™ PCR DNA and Gel Band Purification Kit, Amersham Biosciences) and then digested with the restriction endonuclease *Xba*I (Fermentas) to obtain smaller fragments. Restriction fragments up to 3 kb were A-tailed using a *Taq *DNA polymerase (Fermentas), cloned into pGEM-T Vector (Promega), and sequenced with universal vector primers. Longer fragments were completed by primer walking. Peripheral regions of the linear mtDNA were amplified via step-out PCR [37], single primer PCR [38], and uneven PCR [39]. *Cox1 *fragments were amplified with primers and PCR conditions described in [35].

### Genome annotation and sequence analysis

Sequences were manually checked and assembled using BioEdit v. 7.0.5.3 [40] and then annotated with ARTEMIS v. 9 [41]. Open reading frames of > 60 aa length were compared to GenBank entries using the BlastP program [42]. Additionally, the corresponding nucleotide sequences and intergenic spacers supposed to contain the RNA genes were manually aligned to the mitochondrial genome of *P. tetraurelia *[GenBank:NC001324]. The tRNAscan-SE server [43] was employed to assign the tRNA genes. The Ka/Ks_Calculator [28] was used to estimate the selective pressure on protein-coding genes. Phylogenetic analyses were carried out with MEGA4 [44]. Nucleotide composition, codon usage, and sequence statistics were analyzed with MEGA4 and different programs implemented in the Sequence Manipulation Suite [45]. The effective number of codons (*Nc*) was estimated with the program CodonW v. 1.3 http://codonw.sourceforge.net// and the number of rare codons was calculated according to [8]. *Nc *describes to what degree the codon usage in a genome is biased. It is a number between 20 and 61 where 20 means only one codon is used for each amino acid and values approaching 61 suggest equal usage of synonymous codons [46].

## Authors' contributions

DB conducted the experiments and performed all necessary analyses, TUB wrote the grant proposal and assisted in the design of the experiments. Both authors wrote the manuscript and approved the final version.

## Supplementary Material

Additional file 1 **Codon usage and open reading frames in *Paramecium***. Effective number of codons Nc in protein-coding genes and putative ORFs of *Paramecium tetraurelia *and *P. caudatum *mtDNA. Click here for file

Additional file 2 **Amino acid composition of the mitochondrial genome**. Amino acid composition in the mitochondrial genomes of *Paramecium caudatum*, *P. aurelia *and *Tetrahymena pyriformis *(values are given in percent). Click here for file
