# Supplementary material for: The mitochondrial genome sequence of the ciliate Paramecium caudatum reveals a shift in nucleotide composition and codon usage within the genus Paramecium
Source: BMC Genomics. 2011 May 31;12:272. doi: 10.1186/1471-2164-12-272 (PMC3118789; doi:10.1186/1471-2164-12-272)
Supplement: Additional file 1 — Codon usage and open reading frames in Paramecium. Effective number of codons Nc in protein-coding genes and putative ORFs of Paramecium tetraurelia and P. caudatum mtDNA. [file 1471-2164-12-272-S1.PDF]

Additional file 1, Table S1

Gene	*Nc* *P.t.*	*Nc* *P.c.*	ORF	*Nc* *P.t.*	*Nc* *P.c.*
cob	44.22	33.27	*ymf56*	43.62	27.46
cox1	53.70	33.47	*ymf57*	51.08	44.20
cox2	50.91	33.23	*ymf59*	41.38	34.77
atp9	43.61	29.92	*ymf61*	39.89	33.46
yejR[a]	41.86	29.64	*ymf63*[a]	51.83	35.67
nad1_a	56.75	36.10	*ymf64*[a]	50.69	34.67
nad1_b	48.37	34.50	*ymf65*[b]	-	-
nad2	60.22	26.95	*ymf66*[b]	-	-
nad3	43.10	33.78	*ymf67*[b]	-	-
nad4	51.37	34.19	*ymf68*	50.34	37.58
nad4L	37.51	27.02	*ymf76*[b]	-	-
nad5	49.78	32.14	*ymf78*	54.26	35.12
nad6	52.82	33.04	*ymf79*	61.00	40.01
nad7	56.77	32.84	*ymf80*	54.27	28.85
nad9	36.70	36.90	*ymf83*	46.67	43.81
nad10	52.43	29.16	*ymf84*	57.77	31.83
rps3[a]	51.21	38.93	*ymf87*[c]	-	-
rps12	56.94	34.91			
rps13	56.39	33.87			
rps14	38.14	29.56			
rps19[d]	55.19	30.48			
rpl2	51.72	33.72			
rpl6	41.78	37.04			
rpl14	52.36	37.80			
rpl16	49.92	30.40			

[a] *yejR*, *rps3, ymf63* and *ymf64* were considerably longer in the mtDNA of *P. caudatum*. For comparative analyses the sequences were adjusted to the gene length in *P. tetraurelia*

[b] *ymf65*, *ymf66*, *ymf67* and *ymf76* each appear to be equivalent to two adjacent ORFs in *P. tetraurelia*. Comparative data are omitted

[c] *ymf87* was only found in *P. caudatum*

[d] *rps19* in *P. tetraurelia* was annotated during the present work
