# Supplementary figures and images for: The mitochondrial genome sequence of the ciliate Paramecium caudatum reveals a shift in nucleotide composition and codon usage within the genus Paramecium
Source: BMC Genomics. 2011 May 31;12:272. doi: 10.1186/1471-2164-12-272 (PMC3118789; doi:10.1186/1471-2164-12-272)

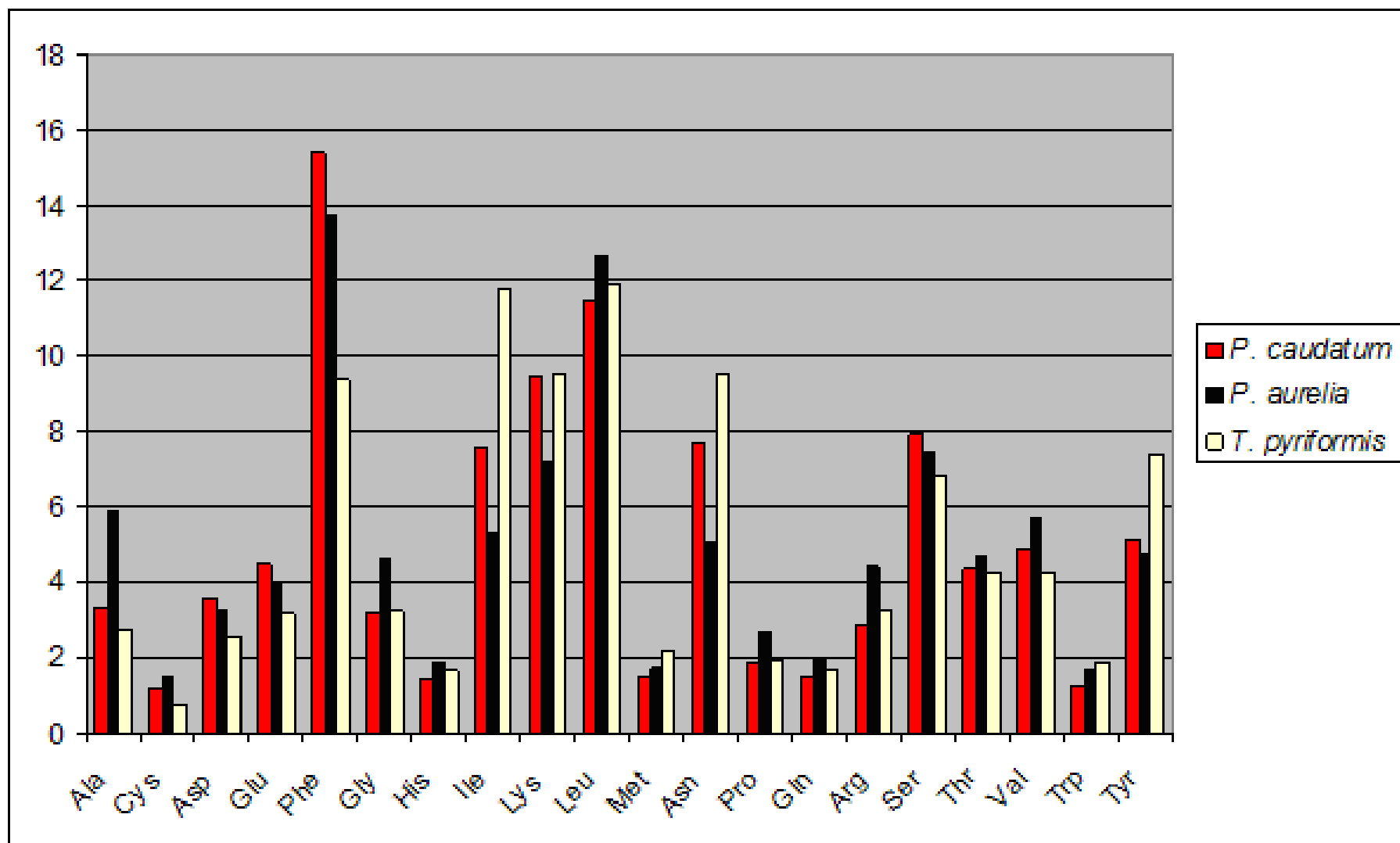

Supplement: Additional file 2 — Amino acid composition of the mitochondrial genome. Amino acid composition in the mitochondrial genomes of Paramecium caudatum, P. aurelia and Tetrahymena pyriformis (values are given in percent). [file 1471-2164-12-272-S2.PDF]
